# Late Stage Infection in Sleeping Sickness

**DOI:** 10.1371/journal.pone.0034304

**Published:** 2012-03-27

**Authors:** Hartwig Wolburg, Stefan Mogk, Sven Acker, Claudia Frey, Monika Meinert, Caroline Schönfeld, Michael Lazarus, Yoshihiro Urade, Bruno Kilunga Kubata, Michael Duszenko

**Affiliations:** 1 Institute of Pathology and Neuropathology, University of Tübingen, Tübingen, Germany; 2 Interfaculty Institute of Biochemistry, University of Tübingen, Tübingen, Germany; 3 Institute of Evolution Biology of Invertebrates, University of Tübingen, Tübingen, Germany; 4 Department of Molecular Behavioral Biology, Osaka Bioscience Institute, Osaka, Japan; 5 African Union/New Partnership for Africa's Development Agency Regional Office for Eastern and Central Africa, Nairobi, Kenya; Univ. Georgia, United States of America

## Abstract

At the turn of the 19^th^ century, trypanosomes were identified as the causative agent of sleeping sickness and their presence within the cerebrospinal fluid of late stage sleeping sickness patients was described. However, no definitive proof of how the parasites reach the brain has been presented so far. Analyzing electron micrographs prepared from rodent brains more than 20 days after infection, we present here conclusive evidence that the parasites first enter the brain via the choroid plexus from where they penetrate the epithelial cell layer to reach the ventricular system. Adversely, no trypanosomes were observed within the parenchyma outside blood vessels. We also show that brain infection depends on the formation of long slender trypanosomes and that the cerebrospinal fluid as well as the stroma of the choroid plexus is a hostile environment for the survival of trypanosomes, which enter the pial space including the Virchow-Robin space via the subarachnoid space to escape degradation. Our data suggest that trypanosomes do not intend to colonize the brain but reside near or within the glia limitans, from where they can re-populate blood vessels and disrupt the sleep wake cycles.

## Introduction

Human African Trypanosomiasis (HAT) has been documented at least since medieval times (13^th^ century) during which its characteristic symptoms (especially sleepiness during daytime) were described by Arabian historians [1]. Descriptions of HAT cases are also well known from times of the slavery tragedy [2] and the first recorded disastrous outbreak of this disease between 1896 and 1906 [3]. Ever since, epidemic outbreaks of this plague were monitored at irregular intervals. Periods of delusive safety always lead to the impression of a nearly eradicated disease. However, although the last outbreak, especially in the Democratic Republic of Congo and Uganda [3], is only some 15 years ago, the currently available drugs (suramin, melarsoprol, pentamidin, eflornithine and recently the combination of nifurtimox and eflornithine) are at least 45 years old and far from being a safe, easy to use, cost-effective and specific treatment, leaving aside the development of resistant strains [4].

HAT is characterized by an early blood and a late brain stage. The blood stage has been extensively documented and is well understood [5]. During this stage, the parasites are protected from the host's immune response by expressing a surface coat consisting of about 10^7^ identical protein molecules known as variant surface glycoprotein (VSG). Since VSG specific antibodies appear in about 10 day intervals, the cell density of parasites in blood is primarily controlled by removal of opsonized trypanosomes, leading to cyclical waves of infection. However, because of antigenic variation [6], [7], HAT is never completely controlled and inevitably fatal if untreated. Amongst the more than 100 trypanosome species, only *Trypanosoma brucei gambiense* and *T.b. rhodesiense* are known to cause chronic or acute form of sleeping sickness, respectively. In the first case it takes months to years, in the latter case weeks to months before parasites appear in brain and induce typical sleeping sickness symptoms such as drowsiness at daytime and insomnia at night, headache, fever, general malaise, slurred speech and changes of personality [8]. When untreated, patients die usually by emaciation, uncontrolled bacterial infection, sometimes acute brain inflammation, but most commonly heart failure [9], [10].

David Bruce was the first to report that *Trypanosoma brucei* are transmitted by tsetse flies [11], before Aldo Castellani found the protozoan parasite within the cerebrospinal fluid (csf) of sleeping sickness patients [12]. However, phylogenetic gene comparisons have shown that salivarian trypanosomes emerged already some 300 million years ago and became midgut parasites of insects [13]. There is no obvious reason why trypanosomes enter the brain of infected mammals, because here they cannot be taken up by tsetse flies during a blood meal. In terms of an evolutionary pressure, one may consider that parasites within the brain are hidden from the major parts of the immune system as a reservoir in case of an eradication of blood parasites. It seems also plausible to assume that sleepiness during daytime increases the chances of trypanosomes to be transmitted to the insect. Tsetse flies are pool feeders, i.e. they cut the skin and suck up blood from the respective lesion. This bite is rather painful and sufficient feeding takes time. Since man can reach virtually any part of the body with his hands, feeding would be more effective if the respective person does not react. Interestingly, prostaglandin D_2_, which induces non-rem sleep if injected into the ventricle system [14], is significantly increased in csf of late stage patients [15]. Trypanosomes are able to produce PGD_2_ [16] that they also use for cell density regulation [17]. During evolution, infection of the brain may thus have proven as an advantage for the parasite's epidemiological distribution.

The way how trypanosomes enter the brain is still controversially discussed. Two possible gates exist, the blood-brain barrier (BBB) and the blood-csf barrier (BCB). Data and arguments have been presented for both options [18], [19], although it is now generally believed that trypanosomes do initially cross the BCB. While in human infections the parasites appear in brain with a latency of at least weeks, in experimental lab infections of rats or mice more than 20 days are needed to detect trypanosomes there [19], [20], [21]. During that time, several waves of high and low parasitaemia have passed where the seal proofed tight. Obviously, it is thus not simply a question of parasite density in blood that pushes the door open but the physiological condition, which may change in time to allow access. The BBB is formed by endothelial cells connected by tight junctions and lined up along a basal lamina of the brain's blood vessels. On the brain side of the vessel, astrocyte feet attach also to the basal lamina and form an additional cellular layer of the BBB [22], the *glia limitans*. In contrast, the BCB does not contain a *glia limitans* and consists of two spatially isolated cellular barriers: 1) Fenestrated endothelial cells line out the capillaries of the choroid plexus and attach to a basal lamina; 2) Epithelial cells forming the surface of the choroid plexus [23]. The latter cells are also based on a basal lamina and are interconnected by a specific type of tight junctions containing claudin 11 [24]. Stroma, i.e. the space between the fenestrated endothelial cells and the plexus epithelia cells, contain connective tissue cells and phagocytes. By passing through the four choroid plexuses, cell-free blood becomes csf that is finally released into the ventricles. The lateral ventricles are connected via the interventricular foramen to the third ventricle and via the cerebral aqueduct to the fourth ventricle. From here, pulsating currents forward csf to the subarachnoid space and the spinal cord.

We here show that trypanosomes are able to cross the BCB and can be located 20 days *post infectionem* (*p.i.*) within the stroma of the choroid plexus as well as inside ventricles. In contrast, we could not detect parasites in the surroundings of blood vessels belonging to the BBB, although they were clearly visible within these vessels. Based on our experimental data obtained so far, we show that African trypanosomes enter the brain via the choroid plexus and settle initially in the pia mater area.

## Results

### Brain infection following intrathecal injection

We used trypanosomes of the pleomorphic strain AnTAT 1.1 and Wistar rats as animal models. To induce a brain infection, animals were either intraperitoneally infected or subjected to an intrathecal injection of parasites. For the latter, 10^4^ trypanosomes in 5 µl were injected either into the striatum (7 rats) or into one lateral ventricle (7 rats). The infected rats have then been observed for 10 days (10 rats) or 14 days (4 rats), respectively, and monitored for behavior and blood infection. Afterwards, isolated brains have been analyzed for parasite distribution. The results of these experiments showed that although the glass capillary orifice was clearly visible by microscopic analysis, trypanosomes could not be detected in the vicinity of this area or elsewhere in the brain. Furthermore, observation of the animals showed no obvious disturbances in activities such as phenotypic behavior, changes in food intake or locomotion (data not shown). Interestingly, no animals that received trypanosomes into the striatum showed a blood infection during the observed experimental time (10 or 14 days), while 6 out of the 7 infected rats that received trypanosomes into the ventricle showed a blood infection 3 or 4 days *p.i.*, as confirmed by tail biopsy. These results clearly suggest that bloodstream form trypanosomes cannot simply enter the parenchyma or csf to induce a brain infection. A retro infection of blood from csf, however, is likely to occur, although we cannot rule out the possibility that during ventricle application in contrast to striatum application blood vessels had been injured.

### Brain infection after blood injection

In nature brain infection occurs only after a prolonged time of blood infection. To analyze brain infection in the animal model, rats were infected by intra-peritoneal injection of 10^7^ trypanosomes in 1 ml citrate-glucose anticoagulant (CGA). Blood parasitaemia was monitored daily by tail bleeding and brains of infected rats were isolated from sacrificed animals between day 5 and 35 *p.i.*

**Figure 1:**
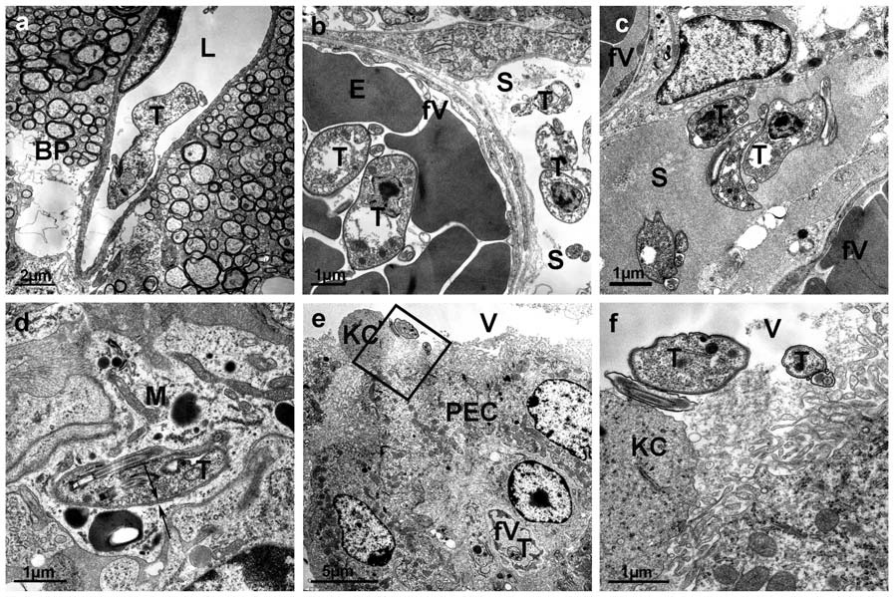
Electron micrographs showing the distribution of trypanosomes in the choroid plexus and ventricle system more than 20 days after a blood infection. **a,** A representative micrograph showing a trypanosome (T) within the lumen (L) of a brain microvessel; no trypanosomes were found beyond the blood-brain barrier within the brain parenchyma (BP). **b,** Cross-section of the choroid plexus showing a fenestrated blood vessel (fV) and trypanosomes (T) within lumen and stroma (S); E, erythrocyte. Trypanosomes inside the blood vessel contain the VSG coat, while parasites in stroma are naked. **c,** Trypanosomes (T) in the stroma (S) between fenestrated capillaries (fV) containing the VSG surface coat. Note: in Figs a–c the brain was directly fixed by perfusion with glutaraldhyde prior to isolation. **d,** A trypanosome (T) inside a plexus cell within a lysosome suggesting phagocytosis of coatless trypanosomes. Trypanosomal and lysosomal membranes are labelled by double arrows. **e,** Low magnification of a choroid plexus showing the ventricle (V), plexus epithelial cells (PEC) and, in the stroma, a fenestrated capillary (fV) with a trypanosome (T). KC = Kolmer cell. **f,** Detail of the apical region of a plexus epithelial cell showing two trypanosomes (T) in close proximity of the microvilli of an epithelial cell and a Kolmer cell (KC), respectively.

**Figure 2:**
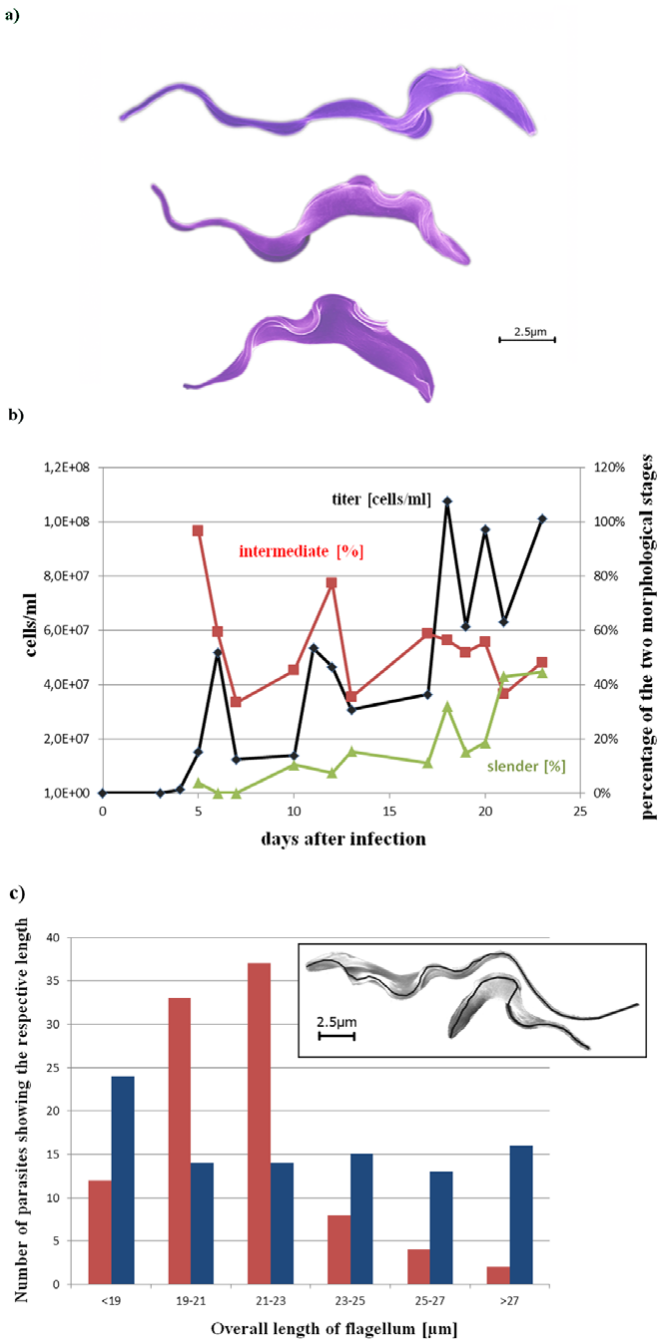
Changes of trypanosomal morphology during the course of infection. **a,** Scanning electron micrographs of typical stumpy, intermediate and slender form trypanosomes. The size of the pictured trypanosome is 17 µm, 22 µm and 26 µm, respectively. **b,** Blood samples of 3 infected rats were analyzed for trypanosome titer up to 20 days (♦ [cells/ml]), shown is a representative curve. Additionally, the overall length of the parasites (n = 27) was measured and grouped into stumpy forms (up to 17 µm), intermediate forms (17–23 µm) and slender forms (longer than 23 µm). Intermediate forms (▪ [%]) represent proliferating trypanosomes. Slender forms (▴ [%]) increase continuously at low level during the course of infection and reach up to 40% after 20 days *p.i*. Distribution of stumpy forms is not explicitly shown but can be calculated as the difference to 100%. **c,** Comparison of trypanosomes isolated from blood or brain of infected rats. AnTat1.1 has been isolated from rat blood (4 days *p.i.*, red bars) and rat brain (35 days *p.i.*, blue bars). The overall length of parasites (n = 96) was measured from the back of the trypanosome to the tip of the flagellum (see inset). We here show that the brain isolate contains a significant amount of trypanosomes longer than 23 µm.

Although parasites were visible within blood vessels at all times, trypanosomes were never detected in the parenchyma outside the vessels (Fig. 1a). This was completely different in case of the plexus area. Comparing peak population densities and brain appearance, we confirmed earlier observations [19], [20], [21] that it takes about 20 days (corresponding to about 3 parasitic waves) until trypanosomes appear in the choroid plexus, independent of the parasite's titer. Whenever the brain was isolated after 20 days *p.i.* or later, trypanosomes, but no blood cells, were clearly visible inside the plexus, i.e. within stroma (Fig. 1b). Thus undoubtedly trypanosomes are able to cross the endothelial cell layer and the underlying basal lamina. Since it is not a question of the number of parasites in blood, the latency of plexus infection cannot easily be explained. To further explore this latency, we isolated brains from infected rats 35 days p.i. to analyze the parasite's morphology. In this case, the brains were isolated without fixation and macerated in media to release life parasites. Interestingly, beside the regular slender and stumpy forms we knew from blood, we observed a very slim and highly moveable form, clearly different from morphological stages seen in blood samples before (Fig. 2a). To further characterize this form, we prepared these brain isolates for scanning EM and counted the length of all morphological stages in 100 SEM micrographs to obtain a statistically relevant impression of the length distribution (Fig. 2b). For this purpose, Olympus cell∧F software was applied to measure the correct length of the flagellum. From the results we considered trypanosomes below 17 µm µm as stumpy, 17–23 µm as slender and above 23 µm as a characteristic slim form from brain. To our surprise, however, this form were also visible in blood, but appeared with a considerable latency (Fig. 2c). Since this correlates very well with the time of plexus appearance, we questioned whether this morphological stage is needed for brain infection. For this purpose rats have been intraperitoneally infected with donor blood from an infected rat 28 days p.i. In this case, trypanosomes were detected within the pial compartment 8 days after infection (data not shown). This result indicates that either the slim morphology differentiates in blood from slender parasites, or it is formed in brain and appeared in blood analogously to a relapse. To investigate whether the accumulation of immune complexes can promote plexus infection, rats were immunized against isolated VSG 221 (Type MITaT 1.2). After 40 days the existence of anti-VSG antibodies was demonstrated by Western blot analysis (data not shown). To induce formation of antibody-antigen complexes, rats were narcotized and 1 mg VSG was injected intravenously over a period of 15 min. Afterwards, the rats were infected with AnTaT 1.1 (which expresses an immunologically different surface coat than the strain used for immunization). In this case, no difference in brain infection was observed compared with control animals receiving an i.p. infection. The result suggests that concentrated immune complexes do not trigger brain infection as a sole factor.

Since stroma contains plasma on its way to become csf, substrate concentrations for e.g. glucose, amino acids, lipids and even protein are very similar or identical to blood and the parasites should find ideal conditions for growth. In contrast, growth inside the choroid plexus seems rather limited, as judged from the population density. Within stroma, we found trypanosomes covered with VSG (Fig. 1c), but also naked parasites without coat (Fig. 1b). Occasionally, we found coatless parasites within a stroma cell (Fig. 1d). It is possible that either unspecific proteases are present in stroma or that the coat is cleaved off by a trypanosomal protease like MSP (see below). Alternatively, trypanosomes may multiply, but because of the high local parasite concentration the prostaglandin D_2_ concentration increases and induces apoptosis, as described earlier [25], [26], [27]. In addition to its stroma location, we detected parasites also within the ventricle, usually in close vicinity to either epithelial cells or Kolmer cells [23] (Fig. 1e). The number was low, but one should keep in mind that free swimming trypanosomes within the ventricle would have been lost together with the csf during preparation of the choroid plexus. In any case, the results show that trypanosomes are able to cross not only the fenestrated endiothelial cell layer but also the epithelial cell layer and to enter the ventricle. Parasites within the ventricle looked healthy and contained the VSG coat.

In principle, csf is a blood filtrate containing usually the same constituents as serum, mostly in a lower, sometime in a higher concentration [28]. Therefore, despite the fact that glucose, the main carbon source for trypanosomal energy metabolism, is reduced by 30 to 50% as compared with blood, csf should support survival of trypanosomes perfectly well.

### Csf as culture medium for bloodstream trypanosomes

**Figure 3:**
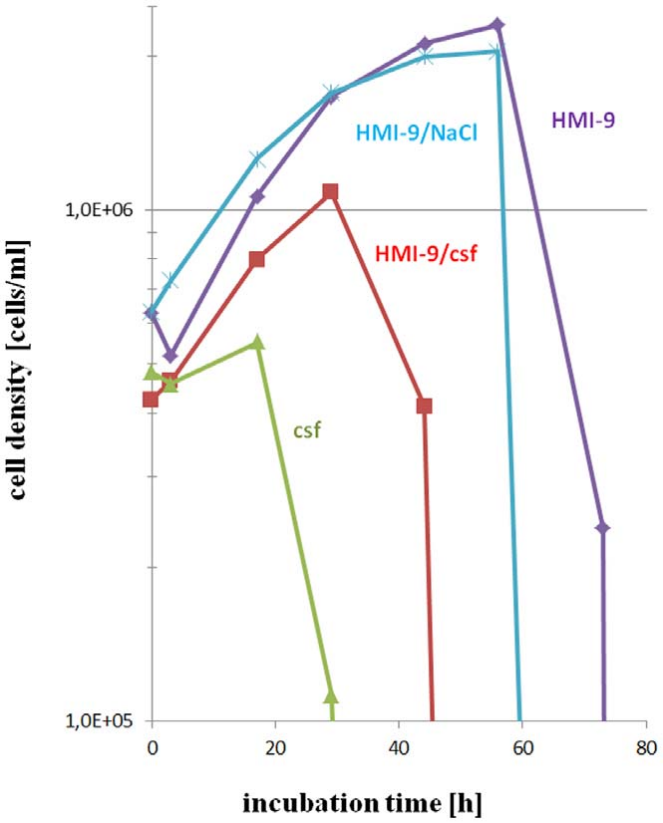
Comparison of trypanosome survival times in cerebrospinal-fluid and/or HMI-9 medium. AnTat1.1 was isolated 11 days *p.i.* from rat blood, separated from blood cells and adjusted to a cell density of 5*10^4^ parasites in 100 µl of the respective solution. Contamination of csf with blood did not exceed 20%, as judged from the erythrocyte count. *Rattus norvegicus* csf supported survival of the parasites only for some 30 hours (▴) and could not be prolonged by supplementing 33 mM glucose (data not shown). However, in a mixture of csf and HMI-9 medium (1∶1), trypanosomes survived significantly longer (i.e. approx. 45 h, ▪). As HMI-9 medium (**♦**) contains all nutrients in excess, it supports growth of trypanosomes for approximately 56 h, even if diluted with saline solution (1∶1, X).

We used isolated csf from different sources: 1) human csf was obtained with patients consent from hospital as surplus materials removed for other purposes; 2) rat csf (400 µl) was obtained from an animal sacrificed because of a hydrocephalus; 3) csf (up to 150 µl per rat) was obtained from sacrificed rats [29]. In the first 2 cases clear csf was obtained, while in the latter case blood contamination could not be avoided, but did not exceed 20% of the volume. These rats had been sacrificed by CO_2_ treatment to avoid a possible contamination with barbiturates. In any case, trypanosomes could not grow in csf. Instead, the cell number decreased slowly within the next 10 h and rapidly thereafter. Addition of 33 mM glucose showed no effect (data not shown) while addition of 50% HMI-9 medium [30] increased survival time to about 50 h (Fig. 3).

In contrast, culture medium diluted 1∶1 with Ringer solution had no impact on trypanosome survival as compared with undiluted medium, obviously because it contains an excess of all necessary components (Fig. 3). This result suggests that in csf a constituent is toxic to trypanosomes, rather than that an essential component is missing. As compared with control cells in culture medium, trypanosomes cultured in csf showed a reduced movement, looked unhealthy in the microscope and underwent necrotic cell death.

### A place for trypanosomes to hide out

**Figure 4:**
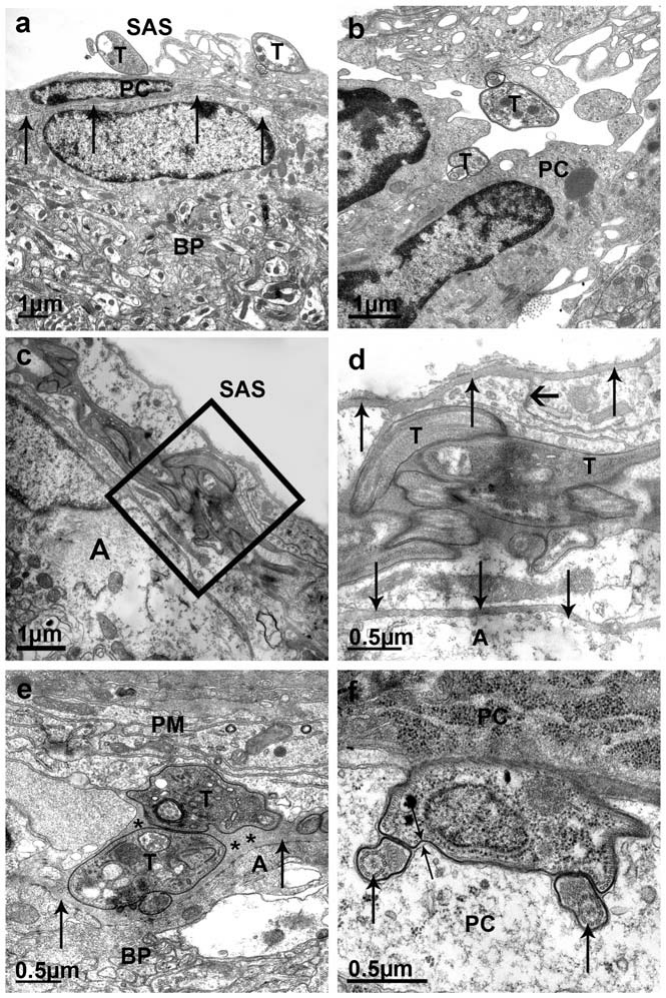
Electron micrographs showing the location of trypanosomes more than 20 days after blood infection. **a,** Trypanosomes (T) are in close proximity to pial cells (PC) within the subarachnoid space (SAS); BP brain parenchyma. **b,** Trypanosomes (T) are located intimately between pial cells (PC) at the intersection between subarachnoid space and *pia mater*. **c,** Area of the *pia mater* containing densly packed trypanosomes between pial cells. A, astroglial endfeet forming the *glia limitans*; SAS, subarachnoid space. **d,** Detail of c. Astrocytes (A) forming the *glia limitans* (lower line of vertical arrows). The upper line of vertical arrows marks the mesothelium. The horizontal arrow labels a tight junction between two pial cells. Trypanosomes (T) are seen between pial cells. **e,** *Glia limitans* (labelled by two arrows) marks the border between brain parenchyma (BP) and *pia mater* (PM); A, foot of an astrocyte. In this image trypanosomes (T) are located within the dilated *glial limitans* (asterisks). **f,** A trypanosome located between pial cells (PC). The two arrows point to the two flagella proving that the parasites are capable of cell division at this location.

Since our experimental data revealed that trypanosomes cannot settle permanently in the plexus or in csf, we considered it likely that they move with the csf current to the subarachnoid space and/or the Virchow-Robin space (VRS). Underneath the cranium three linings of the brain are found: *Dura mater, arachnoidea mater and pia mater*. The space between *arachnoidea* and *pia mater*, the subarachnoid space is completely filled with csf. The VRS is an extension of the subarachnoid space that surrounds blood vessels emerging from arachnoid vessels to enter the brain. Thus the VRS follows the blood vessel for a short distance and ends blindly; its wall is formed from *pia mater* [31], [32], [33]. Analyzing the subarachnoid space and VRS by electron microscopy studies, we easily detected large numbers of trypanosomes within the *pia mater* that consists of up to six pial cell layers and the extracellular matrix [34]. Our EM micrographs clearly show that trypanosomes are found in the subarachnoid space (Fig. 4a), from where they move between pial cells (Fig. 4b) and settle there (Fig. 4c). In this area the parasites consistently contained the VSG coat, showed no intracellular peculiarities as compared to the blood stages and were often densely packed (Fig. 4d). We frequently observed dividing stages (Fig. 4f), but never detected intracellular trypanosomes, although VRS is known to contain macrophages and lymphocytes [35]. Pial cells lay on top of the *glia limitans* or moved inside the dilated *glia limitans*, i.e. the border to the brain parenchyma (Fig. 4e). Thus the trypanosomes have not entered the brain, but stayed outside the blood and csf systems, finding access to the foremost border of the brain. From here the parasite's metabolic waste (like pyruvate) should be removed by diffusing to csf. On the other hand, secreted signal molecules like PGD_2_ will limit trypanosome cell density in this limited space, but would also distributed throughout csf to influence sleep-wake behavior [26], [36], [37], [38]. It should be noted that in late stage patients a highly elevated concentration of PGD_2_ was detected previously [15].

## Discussion

We used electron microscopy studies to investigate the consecutive steps of trypanosomal brain infection. Our findings show that trypanosomes first penetrate the BCB while no penetration of the BBB has been detected. Inside the choroid plexus, trypanosomes were localized within the blood capillaries and inside the stroma. Interestingly, some trypanosomes in stroma had no surface coat and showed disintegration of the plasma membrane. Occasionally, we also detected coatless trypanosomes inside stroma cells within a lysosome, but these parasites were obviously phagocytized and not, as suggested earlier [39], an intracellular stage. From our result we assume that stroma is a rather hostile environment and trypanosomes have to move out quickly to survive. This would explain the low density of trypanosomes within the choroid plexus. Why some but not all of the parasites that entered the plexus lost their VSG coat remains speculative, but may just be a matter of time. If a parasite dies in stroma, loss of membrane integrity would activate the GPI-specific phospholipase C to cleave the coat off [40]. In order to survive, trypanosomes have to cross the epithelial cell layer to reach the ventricle. We have not seen a parasite between or inside epithelial cells, but, as judged from their morphological integrity, we found intact trypanosomes inside the ventricle. Our experiments to cultivate trypanosomes in csf clearly revealed that they cannot multiply or survive for long. This observation is consistent with an earlier report [41]. Thus it is rather unlikely that a considerable population density, comparable with blood infection, can be reached in csf. From this perspective it becomes also clear why lumbal puncture has a limited benefit as a diagnostic tool: As published recently [42], the number of trypanosomes in csf obtained by lumbal puncture from HAT patients is usually zero or very low, and thus lymphocytes are often counted as a general marker for inflammation, which together with HAT symptoms are indicative for late stage disease.

For a sustained survival in brain, trypanosomes have to reach a more convenient location, apart from plexus and csf. Our experimental data show that parasites do not enter the brain parenchyma, but reside inside the *pia mater* close to the *glia limitans*. Thus parasites living in this location should be supported by substrates like glucose, amino acids, lipids and nucleosides, while being protected from dangerous components of csf. Unlimited growth of these trypanosomes would certainly be dangerous for the host. However, cell density control by PGD_2_-induced apoptosis [16], [17], [26] limits the risk by avoiding inflammation reactions. In addition, secreted PGD_2_ could easily be distributed via csf and could induce the observed disruption of sleep wake cycles. With time, trypanosomes may find their way across the *glia limitans* to invade the parenchyma. Here, contact with microglia cells could lead to inflammation [43] and eventually cause lymphocytes to enter the brain. It should be noted that in many cases people seem to die prior to this stage, i.e. before encephalitis is observed [19]. We thus hypothesize that breakdown of the BBB at the final stage of brain infection is the result of an inflammation and not induced by the infiltration of parasites from the blood side. Instead of moving forward from its pial location to enter the brain, trypanosomes may also move backwards into csf and further to blood by crossing the arachnoid villi during csf resorption. This could account for relapses occurring after clearance of blood parasites by suramin treatment and may also be the reason for the observed blood infection after intrathecal injection of parasites into the ventricle. We cannot exclude that trypanosomes may also enter the pial compartment from leptomeningeal vessels, but regard this possibility as rather unlikely considering the confirmation of the plexus–ventricle route.

To our knowledge, it has so far not been attempted to apply trypanocidal drugs (like suramin or pentamidin) by intrathecal injection using lumbal puncture. Considering the parasites location in the pial and subpial compartment, it might be worthwhile to explore this possibility that is successfully used for intrathecal chemotherapy of brain tumor patients, to treat HAT.

The question remains why trypanosomes cross the BCB but not the BBB. An explanation in this respect may come from the constitution of the BBB versus BCB. Usually, the BBB cannot be penetrated by cells while biochemical compounds need specific carrier proteins. It is known that in case of inflammation lymphocytes are able to cross the BBB, but in this case specific cell-cell interactions are indispensable to open the gate temporarily [44]. It has been discussed that trypanosomes could just follow lymphocytes or that they can open the BBB temporarily using small molecules like γ interferon [45]. Both possibilities seem unlikely to us. Lymphocytes perform a highly sophisticated strategy to penetrate either para- or trans-cellular [46], but in any case, one lymphocyte enters as an individual cell, while the gap is closed afterwards. Also, for a trypanosome to follow a lymphocyte, a brain inflammation must have been occurred previously due to another event like injury or a different infection. The small molecule theory suggests that a high local concentration of parasites within the blood vessels would induce themselves or host cells to secrete this molecule, rendering it difficult to explain the latency of brain infection. Also, existence of a parasite specific factor to open the BBB has not been substantiated so far.

In terms of the BCB, the situation is different. Here blood vessels are lined with fenestrated endothelial cells, while a *glia limitans*, composed from a basal lamina and attached astrocytes, is missing. Thus the barrier is less dense and blood pressure allows the liquid parts of blood to expand into the choroid plexus. Using flagella movement, trypanosomes may orientate themselves to this current and penetrate. Since flagella movement pulls a trypanosome forward, it is likely that the free end of the flagellum undergoes cell contact with the fenestrated endothelial cells first to open the gate. However, it takes some 20 days of infection before this process happens, and thus some additional factor seems to be needed. It has been described for other diseases [47] and suggested for HAT [48] that antigen-antibody complexes (in this case the VSG-antibody complex) may concentrate inside the plexus, thus leading to inflammation and weakening of the endothelial fenestration. This could indeed account for the observed latency, although one might expect that blood cells would also enter the plexus in this case. In our preliminary experiments, induction of VSG-antibody complexes did not result in a more rapid brain infection, indicating that vsg-antibody complexes do not account for the observed latency. Nevertheless, crossing the blood plexus border is only half of the way to reach csf. The border between plexus and ventricle is built from epithelial cells firmly connected by tight junctions. It has been observed earlier that trypanosomes not only enter the brain but can also be found in testis [49]. The respective blood-testis barrier is formed from Sertoli cells. Interestingly, tight junctions between plexus epithelia as well as Sertoli cells contain claudin-11 [24], which leads to formation of a more parallel orientation of the junctional strands as seen in freeze-fracture replicas, while all other tight junctions show a branched appearance of strands [50]. It is tempting to speculate that trypanosomes may have developed the ability to specifically interact with claudin-11, but this has to be investigated in detail. Both parts of the BCB contain, in addition to the respective cell layers, a basal lamina that has to be penetrated. From the movement of cancer cells during metastasis it is known that metalloproteases are able to open a basal lamina [51]. Since bloodform trypanosomes are able to express suitable metalloproteases (MSPs) [52], the basal lamina may be easily crossed by this parasite. TbMSP-B may also account for the loss of the VSG coat on individual parasites within stroma.

The presented scenario how trypanosomes invade the brain is based on our experimental findings and can explain several observations describing sleeping sickness. It cannot explain, however, why it takes some 20 days in our animal model, or weeks to months in nature, until the parasites enter the choroid plexus. We found a very thin and fast moving slender form that we observed during late peak populations in blood, which was also the predominant form of trypanosomes isolated from brain. It is tempting to speculate that an additional lifecycle stage exists that is especially adopted to live in brain. This would also explain why we did not find a brain infection by intrathecal injection of bloodform trypanosomes into the brain. Nevertheless, we consider the three morphological stages shown in Fig. 2a rather as slender, intermediate and stumpy, leading to the consequence that intermediate trypanosomes would be the major form to support blood infection, while slender parasites are involved in brain infection, and stumpy parasites die either by apoptosis or are opsonized by antibodies and phagocytized by macrophages. This view on the different roles of the 3 morphological stages in blood is supported by the fact that stumpy parasites do not undergo antigenic variation or cell division [53] and that formation of monomorphic strains (induced by an exclusive blood to blood infection of laboratory animals) leads to parasites of an intermediate rather than slender morphology. The question of the physiological function of slender form trypanosomes is currently addressed in our laboratories. One might ask why the infected blood samples used to infect our laboratory animals did not contain slender parasites. The reason is that we prepare stabilates (blood samples to be stored in liquid nitrogen and used for animal infection) always from an infected rat during the first parasitaemic wave, i.e. at a time when slender parasites have not been formed yet, and in nature a mammal infection starts with the metacyclic insect form. Consistent with this considerations, a pial infection appeared before day 10 (instead of day 20), if infection of rats was not performed using regular stabilates but infected blood from rats infected for more than 20 days.

In conclusion we show that part of the trypanosome population escapes from blood by crossing the BCB eventually finding their way to the pial and subpial space. Here, i.e. still outside the brain parenchyma, the parasite finds an ideal place being protected from all means of the immune response with the option to reinvade blood. In well adapted hosts like trypano-tolerant animals, this might be the final destination to ensure a lifelong infection. In suboptimal adapted hosts like human, finally the parasite may cross the *glia limitans* at a later stage of infection, leading to encephalitis and death.

## Materials and Methods

### Ethics Statement

This study was carried out in strict accordance with the German Animal Welfare Act. The protocol was approved by the Regional Commission of Tübingen (Permit Number: IB 3/10). All surgery was performed under ketamin/xylazin anesthesia, and all efforts were made to minimize suffering. For experiments with surplus csf, patients were educated as recommended by the ethics committee at the University Hospital of Tübingen. This committee approved the usage of surplus csf for the presented study. Written informed consent was provided by study participants.

#### Isolation of trypanosomes from blood or brain

Rats were infected intraperitoneally with 5×10^7^ cells, mice with 5×10^6^ cells. The parasite titer was monitored daily by tail biopsy. After defined time periods, animals were narcotized with ketamin (100 mg/kg body weight)/xylazin (10 mg/kg body weight), 1 ml citrate glucose anticoagulant (CGA; 102 mM citrate, 40 mM glucose, pH 7.7) was given into the opened thorax to prevent blood clotting. The vena cava was cut and the escaping blood was kept on ice. Trypanosomes were purified by ion-exchange chromatography [54] and fixed in paraformaldehyde (4% w/v) / glutaraldehyde (4% v/v). Blood circulation was flushed with CGA, brains were dissected, chopped and incubated in 20 ml HMI-9. Tissue was removed by filtration and the trypanosomes fixed as above.

#### Brain isolation

To analyze trypanosomal brain infection, infected animals were narcotized before opening the thorax. Placing a syringe needle, connected to a buffer reservoir, into the left heart ventricle, the vessel system was extensively washed by perfusion with a glucose containing isotonic phosphate buffer that was replaced by a fixative solution containing paraformaldehyde/glutaraldehyde (4% each) thereafter. In some cases, perfusion was directly performed with a glutaraldehyde containing isotonic salt solution, to also fix cells within vessels.

#### Intrathecal Injection

For intrathecal injection narcotized rats were positioned into a stereotaxic instrument (Narishige scientific instruments). The injection site was localized with the aid of a rat brain atlas [55]. Trypanosomes (10^4^ parasites in 5 µl) were injected with a pulsed micropump (Picospritzer III, Parker Hanifin Corporation) at a rate of 250 nl/min. The fixed brain was frozen in tissue medium (NEG 50™, Thermo scientific) and stored at −80°C for cutting frozen sections of 10–30 µm thickness with a cryostat microtome.

#### Transmission electron microscopy

Tissues were post-fixed in osmiumtetroxide (1%) in cacodylate buffer. After three washes with D-PBS the samples were dehydrated by a graded ethanol series (30%, 50%, 70%, 90%, 96% for 15 min each, 2×99% for 30 min each) and two washes with propylenoxide. During the 70% ethanol step of the graded ethanol series, the specimens were incubated in saturated uranyl acetate. After completion of dehydration, the preparations were embedded in Araldite 502 (Sigma-Aldrich) at 60°C for 48 h. Ultrathin sections were prepared on a Leica FCR Ultracut ultramicrotome and stained with lead citrate. Sections were examined using a Zeiss EM 10 electron microscope [56].

#### Scanning electron microscopy [57]

Samples were fixed with 2.5% glutaraldehyde in cacodylate buffer, postfixed with 1% osmium tetroxide in phosphate-buffered saline, dehydrated in a graded series of ethanol and critical-point-dried using CO2. After critical point drying they were fixed on specimen holder stubs, sputter-coated with gold, and viewed with a scanning electron microscopy (SEM) (Cambridge Stereoscan 250 Mk2, Cambridge Scientific, Cambridge, UK).

#### Immunization of rats against variant surface glycoprotein

Rats received initially 100 µg VSG in 100 µl Freund's complete adjuvant subcutaneously and were boosted once after 15 days. After 40 days the existence of anti-VSG antibodies was proven using Western blot analysis.
